# Deep-learning radiomics and hand-crafted radiomics utilizing contrast-enhanced MRI to predict early peritumoral recurrence after DEB-TACE with hepatocellular carcinoma: a two-center study

**DOI:** 10.3389/fonc.2025.1642828

**Published:** 2025-11-18

**Authors:** Jin Wang, Huan Liu, Yiman Li, Xueqin Ma, Hao Chen, Xiaoping Luo, Baolin Zhou, Xi Liu

**Affiliations:** 1 Department of Radiology, The Second Affiliated Hospital of Chongqing Medical University & Chongqing Medical Imaging Artificial Intelligence Laboratory, Chongqing, China; 2 GE Healthcare, Advanced Analytics Team, Shanghai, China; 3 Department of Radiology, The First Affiliated Hospital of Army Military Medical University, Chongqing, China

**Keywords:** hepatocellular carcinoma, DEB-TACE, contrast-enhanced MRI, deep learning, radiomics

## Abstract

**Purpose:**

To investigate early peritumoral recurrence (EPR) after drug-eluting bead transarterial chemoembolization (DEB-TACE) in a multicenter cohort of patients with hepatocellular carcinoma (HCC) using deep learning radiomics (DLR) based on preoperative multiphase magnetic resonance imaging (MRI).

**Patients and methods:**

A total of 157 patients with HCC from two institutions who received DEB-TACE were retrospectively enrolled and divided into a training cohort (n=114) and an external validation cohort (n=43). A total of 960 radiomics features were extracted from five different phases: arterial phase (AP), delayed phase (DP), portal venous phase (PVP), peritumoral 3 mm portal venous phase (PVP_Pri3mm), and tumoral plus peritumoral portal venous phase (PVP_Plus3mm). A total of 512 deep learning features were extracted from PVP using ResNet34 (PVP_DLR). The features selected through the minimum Redundancy and Maximum Relevance (mRMR) and Least Absolute Shrinkage and Selection Operator (LASSO) methods were utilized for model construction. The performance of the model was evaluated using area under the curve (AUC), calibration curves, net reclassification (NRI), and decision curve analysis (DCA).

**Results:**

PVP_Pri3mm and PVP_Plus3mm showed comparable performance to the PVP model (P>0.05). The final deep learning radiomics and radiomics nomogram (DLRRN) included three predictors: PVP-signature, PVP_ DLR signature, and AFP, which showed effectively discrimination of between EPR to DEB-TACE, with AUCs of 0.802 (95% CI, 0.718-0.887) in the training cohort and 0.770 (95% CI, 0.623-0.916) in the external validation cohort, demonstrating good calibration (P>0.05). Additionally, the DLRRN model performed significantly better than the clinical model (P<0.05). DCA confirmed that DLRRN was clinically useful.

**Conclusion:**

DLRRN has good efficacy in predicting EPR after DEB-TACE, which can provide value for preoperative treatment selection and postoperative prognostic assessment of patients with HCC.

## Introduction

Hepatocellular carcinoma (HCC) is primarily linked to chronic liver disease, being the fifth most common malignant tumor worldwide and the second highest contributor to cancer-related deaths (1). Transarterial chemoembolization (TACE) is the main therapy recommended for intermediate-stage HCC according to the Barcelona Clinic Liver Cancer (BCLC) staging system. However, recent findings have demonstrated its effectiveness across various stages of HCC (2). Currently, following the European Association for the Study of the Liver (EASL) guidelines, TACE is well-recognized as a neoadjuvant treatment before liver transplantation, playing a vital role in reducing the tumor burden (3).

Two distinct techniques have been used for TACE. Conventional TACE (cTACE) is the most widely practiced modality globally, utilizing a suspension of lipiodol and chemotherapeutic agents. Alternatively, TACE can be performed by using drug-eluting beads (DEB-TACE) (4). Although some studies have shown that DEB-TACE is superior to cTACE in terms of local tumor control rate, systemic adverse reactions, toxicity, and survival rate, the actual choice of treatment often considers multiple factors due to the clinical heterogeneity of hepatocellular carcinoma. Consequently, accurately predicting treatment response and prognosis through imaging and other methods before the procedure is crucial to selecting the most appropriate treatment.

Radiomics is an emerging technology that uses high-throughput extraction algorithms to quantify features, thereby enabling the more comprehensive and efficient mining and exploitation of information in medical images (5). Radiomics has been applied to predict treatment response (6, 7), recurrence (8) and survival (9, 10) of HCC. In several studies, radiomics has been used to predict response to treatment in HCC, identifying radiomic features that were significantly correlated with response to surgery, radiofrequency ablation, chemotherapy, and TACE therapy (11–15). However, few studies have been reported on the assessed HCC by using radiomics after DEB-TACE. Patients with early peritumoral recurrence have a significantly lower survival rate and a poor therapeutic response to repeated TACE. Additionally, DL features have enabled radiomics to obtain intricate structures related to specific tasks, resulting in excellent results in tumor characterization and prognostic prediction in gastric, breast, rectal, and nasopharyngeal cancers (16–19). In our knowledge, no study has examined the association between deep learning radiomics (DLR) and early peritumoral recurrence prediction in HCC patients.

Therefore, accurate preoperative assessment is vital for the choice of treatment and improvement of postoperative recurrences. The purpose of this study was to assess early peritumoral recurrence (EPR) after DEB-TACE in a multicenter cohort using DLR based on preoperative multiphase enhanced MRI.

## Materials and methods

### Patients population

**Figure 1:**
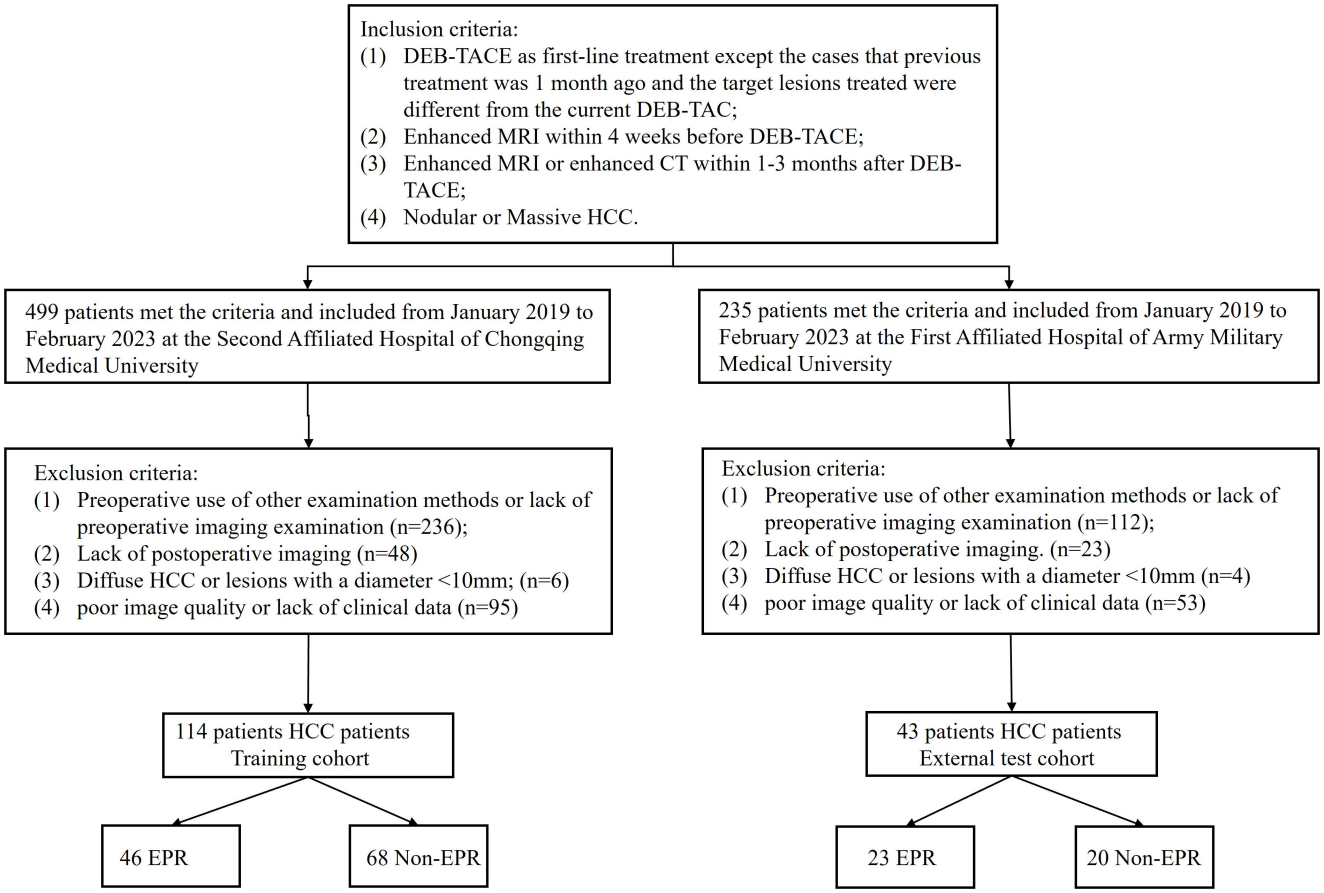
Flowchart of the study population selection.

This retrospective study was approved by the institutional review boards of the two hospitals (2023(145)), and the need for informed consent was waived. A total of 499 patients with hepatocellular carcinoma (HCC) who underwent treatment with DEB-TACE at the Second Affiliated Hospital of Chongqing Medical University, and 235 patients from the First Affiliated Hospital of Army Medical University between January 2019 and February 2023. The inclusion criteria were as follows: 1) DEB-TACE as first-line treatment except the cases that previous treatment was 1 month ago and the target lesions treated were different from the current DEB-TAC; 2) Enhanced MRI within 4 weeks before DEB-TACE; 3) Enhanced MRI or enhanced CT within 3 months after DEB-TACE; 4) Nodular or Massive HCC. First-line treatment was defined as the initial treatment administered to a patient who had not received any prior therapy at the time of their HCC diagnosis. The exclusion criteria were as follows: 1) preoperative use of other examination methods or lack of preoperative imaging; 2) lack of postoperative imaging; 3) Diffuse HCC or lesions with a diameter <10 mm; and 4) poor image quality or lack of clinical data. Ultimately, 114 HCC patients from center 1 served as the training cohort, and 43 patients from center 2 constituted an independent external validation cohort and were included in the study (**Figure 1**).

Routine preoperative clinical characteristics and descriptions of the DEB-TACE procedure are provided in the **Supplementary Material**. All patients were evaluated using multiphase enhanced MRI or CT within 1–3 months after DEB-TACE, with the follow-up endpoint of early peritumoral recurrence, defined as the internal or marginal portion of the lesion that was enhanced in the arterial phase and faded in the venous and delayed phases.

### MRI examination and image preprocessing

All MRI examinations included arterial phase (AP), portal venous phase (PVP), and delayed phase (DP) images, which were obtained at 15–25 seconds, 50–60 seconds, and 150–180 seconds after contrast injection, respectively. Details regarding the MR acquisition parameters of the two centers are presented in (**Supplementary Table S1****).**

### Tumor segmentation

**Figure 2:**
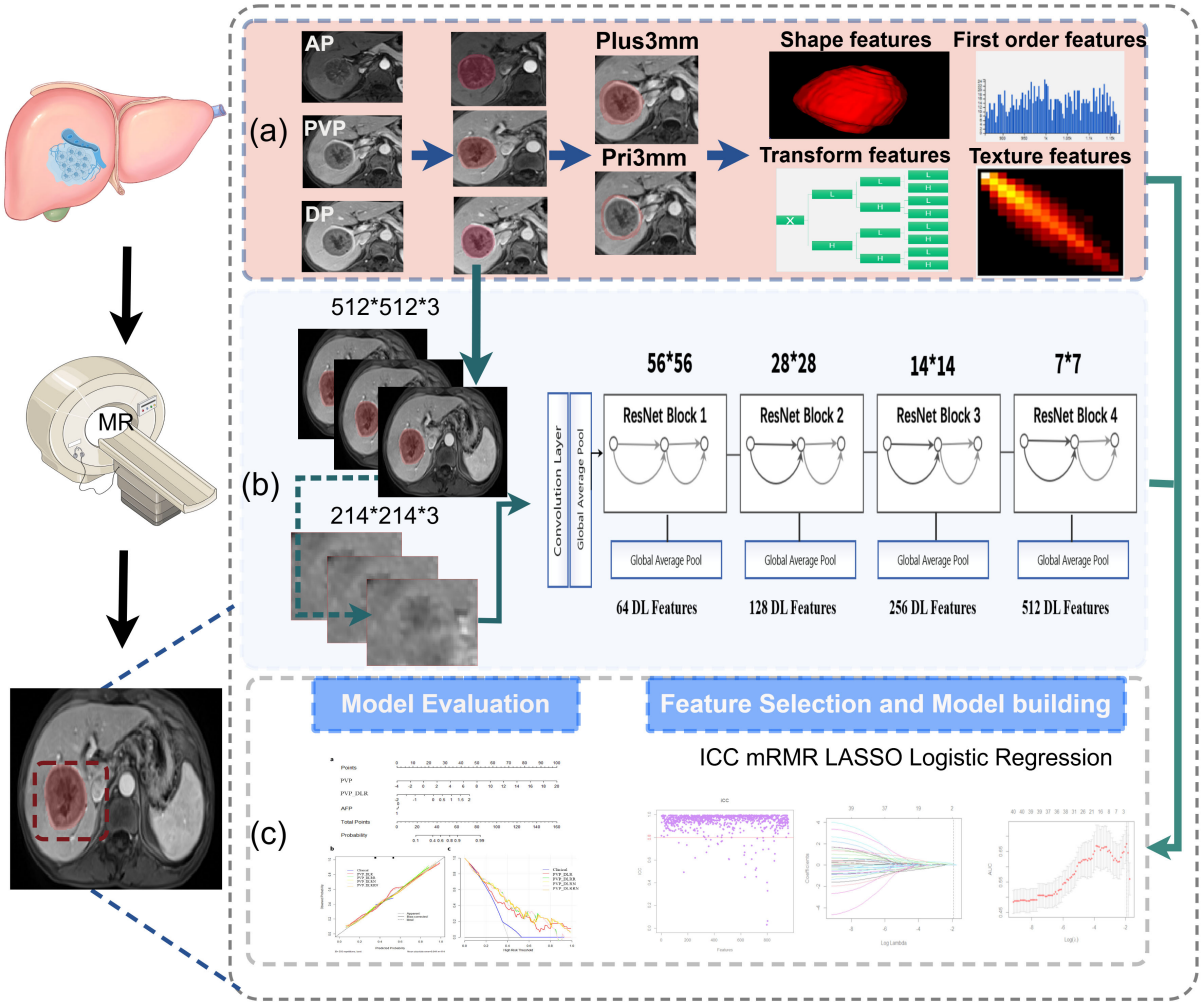
Overall workflow of study. **(a)** Tumors were manually delineated around the entire tumor outline on each axial slice of arterial phase (AP), delayed phase (DP), portal venous phase (PVP) images, and the peritumoral expansion (PVP_Pri3mm), the tumoral plus peritumoral (PVP_Plus3mm) were automatically generated in PVP images; total 960 radiomics features were extracted each volume of interests, respectively. **(b)** The detailed architecture of ResNet34, and the 512 deep features were obtained from PVP images. **(c)** The workflow of feature selection, model building and evaluation.

The imaging data were collected using the picture archiving and communication system (PACS) at the Second Affiliated Hospital of Chongqing Medical University and the First Affiliated Hospital of the Army Military Medical University, and patients’ preoperative multiphase enhanced MRI were exported in DICOM format. The MRI underwent resampling through linear interpolation to achieve a voxel size of 1×1×1 mm3, thereby standardizing the voxel spacing. The region of interest (ROI) was delineated by two radiologists using the 3D-Slicer software (version 4.10.2, https://download.slicer.org), which provides a powerful function for semi-automatic segmentation. Contrast-enhanced magnetic resonance imaging (AP, PVP, and DP) was performed to segment the tumor and avoid the surrounding tumor vessels. To capture features from the 3 mm peritumoral area (ROI-external) in the PVP, which has a higher potential for microvascular invasion, a dilation algorithm was applied to obtain the 3-mm wide area. The combined intratumoral and peritumoral areas (ROI-plus) were generated simultaneously. Importantly, non-hepatic regions within the ROI were subtracted either semi-automatically or manually on a slice-by-slice basis, as appropriate. Ultimately, five ROIs were identified from these three phases after the segmentation process for each patient. (**Figure 2a**).

### Hand-crafted feature extraction

All handcrafted features were extracted utilizing the PyRadiomics package. The voxel intensity values were discretized using a fixed bin width of 5. A total of 960 quantitative features were calculated from each ROI in accordance with the guidelines set by the Image Biomarker Standards initiative (IBSI) (20), including first-order statistical features, shape features, textural features, and transformation features. Statistics-based textural features can reflect the homogeneity of the images and the arrangement of properties that change slowly or periodically on the body surface, including gray-level co-occurrence matrix (GLCM), gray-level run length matrix (GLRLM), gray-level size zone matrix (GLSZM), neighboring gray tone difference matrix (NGTDM), and gray-level dependence matrix (GLDM) features. There were advanced filters applied using the Laplacian of Gaussian (LoG, sigma 1.0 mm) and wavelet decompositions with all possible combinations of high (H) or low (L) pass filters in each of the three dimensions (HHH, HHL, HLH, LHH, LLL, LLH, LHL, and HLL). Detailed information on these features is available in PyRadiomics (http://PyRadiomics.readthedocs.io/en/latest/).

### Deep learning feature extraction

Each slice of the tumor was bound by a cubic bounding box during data preprocessing to ensure that the entire tumor was contained within the bounding box. Then, an area of 224 × 224 pixels containing the tumor was cropped as the final image input for the DL models. ResNet-34 was used to build a signature on MRI (21). Due to the millions of learnable parameters in DL models, training it is computationally expensive and requires a large number of images. With transfer-learning technology, a DL model can be trained on less data. A DL model with ResNet34 architecture was trained using the ImageNet dataset with PyTorch (version 1.4.1; PyTorch. org). In the ResNet34 model, the fully connected and softmax layers were removed, and the output values of the nodes in the last layer were used as DL features. The bounding boxes of the images on three adjacent MRI slices were combined into a three-channel image as DL model input. To achieve robust prediction, all three-channel images of each tumor were fed into the DL model (**Figure 2b**). Based on the DL model, a total of 512 DL features were extracted and selected (**Figure 2**). The average value of the prediction probability of multiple tumor images was calculated as the DL signature.

### Feature selection and signature building

For the training cohort, a four-step procedure was used for feature reduction. First, interobserver correlation coefficients (ICCs) were calculated to explore the stability and reproducibility of features, and only the features with both inter- and intra-ICCs > 0.80 were considered to have agreeable reproducibility and were chosen for further analysis. The abnormal values were replaced by the median, all features were standardized, and z-score normalization of MRI signal intensities was performed to eliminate the variance of features before selection. Second, we removed redundant and less-relevant features using minimum redundancy and maximum relevance (mRMR). Then, the optimized feature subsets were selected using the least absolute shrinkage and selection operator (LASSO) method with 10-fold cross-validation (**Supplementary Figure S1**). Finally, a multivariate logistic regression analysis was performed to build the signature. The radiomic signature was generated using a linear combination of selected features weighted by their respective regression coefficients. The cut-off value was then identified using Youden’s index to divide the patients into non-EPR and EPR subgroups.

### Performance evaluation

There are five radiomics models: the arterial phase model (AP), portal venous phase model (PVP), delay phase model (DP), peritumoral 3 mm portal venous phase model (PVP_Pri3mm), tumor plus peritumoral portal venous model (PVP_Plus3mm), and one deep learning radiomics model based on the portal venous phase (PVP_DLR). Moreover, a clinical model and related combined models, such as the deep learning radiomics and radiomics model (PVP_DLRR), deep learning radiomics nomogram (PVP_DLRN), and deep learning radiomics and radiomics nomogram (PVP_DLRRN) were established. The performance of all established models for HCC recurrence was measured using receiver operating characteristic (ROC) analysis, and the area under the ROC curve (AUC) was calculated and compared among cohorts using the DeLong test. In addition, sensitivity and specificity were measured. The net reclassification index (NRI) was calculated to compare the performance of the models.

### Statistical analysis

Statistical analyses were performed using SPSS (version 26.0, https://www.ibm.com/spss) and R (version 4.2.1, available at http://www.R-project.org). The chi-square test or Fisher’s exact test was used for nominal variables. A logistic regression analysis was performed using the “glmnet” package. The diagnostic performances of the models were compared using ROC analysis, and the differences in the AUCs between these models were compared using the Delong test. Receiver operating characteristic (ROC) curves were plotted using the “pROC” package. All statistical tests were two-sided, and statistical significance was set at P <0.05.

## Results

### Clinical characteristics

**Figure 3:**
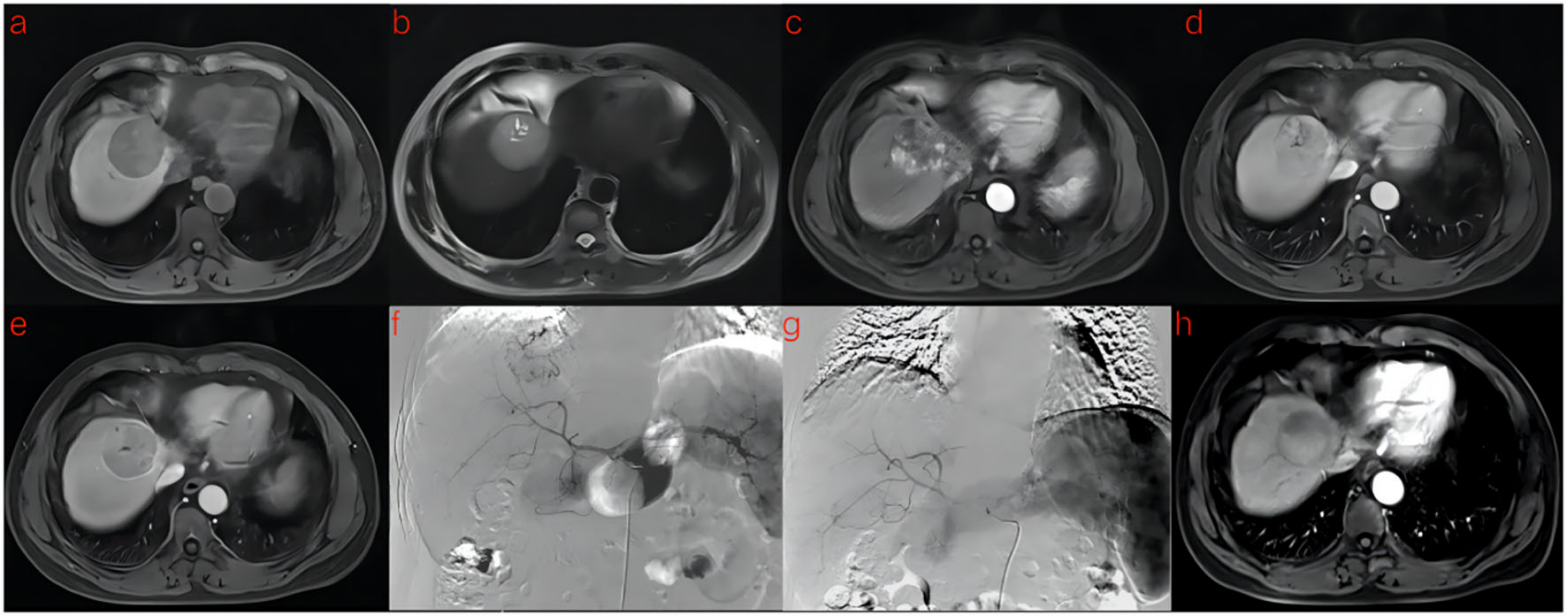
A 53-year-old man with HCC was treated with DEB-TACE. **(a–e)** MR examination revealed that the tumor was located in the segment 4 of the liver. **(f)** The tumor supplying artery was the hepatic arteria 4. **(g)** The tumor supplying artery was embolized by 300-500 μm pirarubicin-loaded beads. **(h)** No abnormal enhancement was found in enhanced MR Lesions after 3 month follow-up.

**Figure 4:**
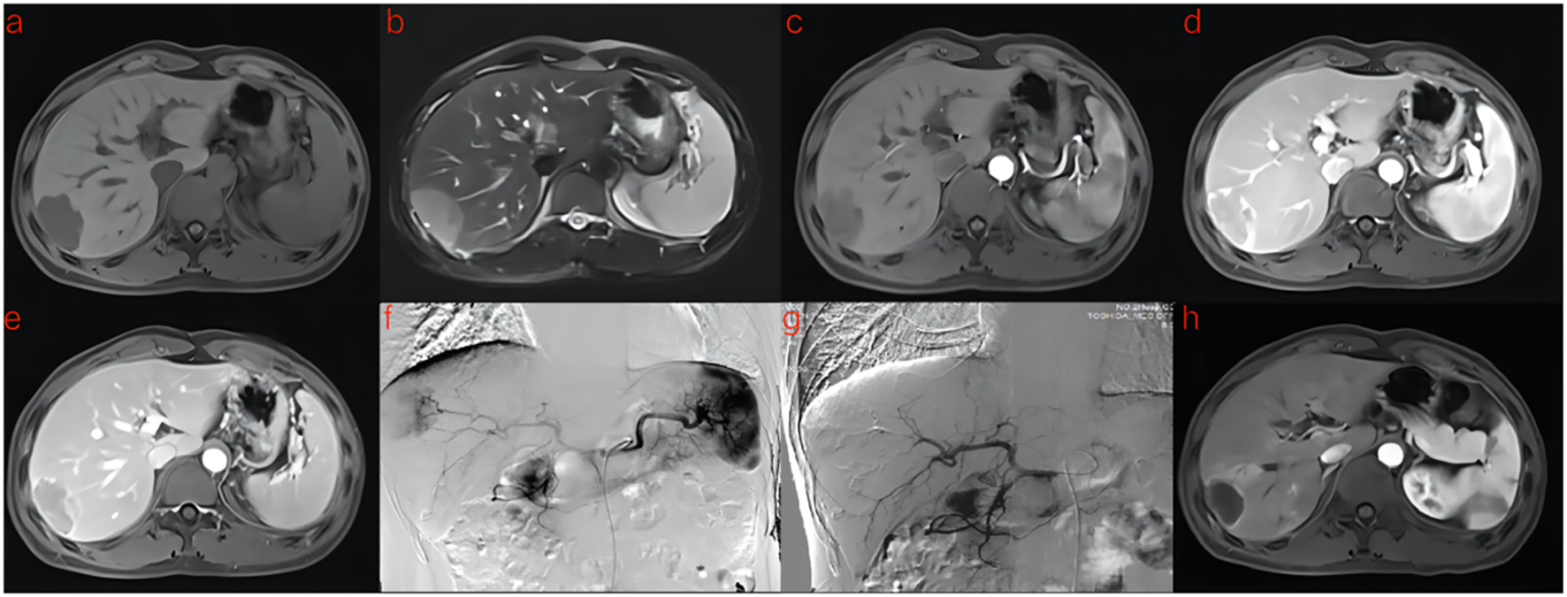
A 41-year-old man with HCC was treated with DEB-TACE. **(a–e)** MR examination revealed that the tumor was located in the segment 7 of the liver. **(f)** The tumor supplying artery was the hepatic arteria 7. **(g)** The tumor supplying artery was embolized by 300-500 μm pirarubicin-loaded beads. **(h)** One months later, MR Enhanced follow-up examination showed abnormal enhanced tumor recurrence.

A flowchart of the study is shown in (**Figure 2**). The baseline clinical characteristics and demographics of the training and validation groups are summarized in (**Table 1**). The enrolled patients were allocated to a training set (n=114) or an external validation set (n=43). The efficacy of DEB-TACE was balanced for patients in the two cohorts, with early peritumoral recurrence rates of 40.3% (n=46) and 46.5% (n=20) for the training and independent external validation cohorts, respectively. Images of the two patients are shown in (**Figure 3**) and (**Figure 4**). No significant differences were detected in sex, age, ALT, ChildPugh, HBsAg, cirrhosis, portal hypertension, tumor number, tumor margin, rim enhancement, or peritumoral enhancement between the recurrence and non-recurrence groups (P>0.05). Moreover, the AFP levels (P = 0.031) were significantly different between the two groups in the training cohort. AST level (P = 0.015), tumor size (P = 0.002), and BCLC stage (P = 0.016) also showed statistically significant differences in the external validation cohort. AFP in the training cohort was constructed for the clinical model (**Table 1**).

**Table 1:** Characteristics of the patients in the cohorts.

Variable	Training cohort (n=114)			External cohort (n=43)			P-value
	Non-EPR	EPR	P-value	Non-EPR	EPR	P-value	
Gender							
0	14 (20.59%)	7 (15.22%)	0.468	2 (8.70%)	6 (30.00%)	0.162	0.979
1	54 (79.41%)	39 (84.78%)		21 (91.30%)	14 (70.00%)		
Age							
0	15 (22.06%)	11 (23.91%)	0.817	8 (34.78%)	5 (25.00%)	0.486	0.337
1	53 (77.94%)	35 (76.09%)		15 (65.22%)	15 (75.00%)		
AFP							
0	54 (79.41%)	28 (60.87%)	0.031*	19 (82.61%)	15 (75.00%)	0.813	0.364
1	14 (20.59%)	18 (39.13%)		4 (17.39%)	5 (25.00%)		
ALT							
0	55 (80.88%)	38 (82.61%)	0.816	21 (91.30%)	15 (75.00%)	0.303	0.755
1	13 (19.12%)	8 (17.39%)		2 (8.70%)	5 (25.00%)		
AST							
0	42 (61.76%)	22 (47.83%)	0.141	13 (56.52%)	4 (20.00%)	0.015*	0.063
1	26 (38.24%)	24 (52.17%)		10 (43.48%)	16 (80.00%)		
ChildPugh							
1	58 (85.29%)	39 (84.78%)	0.94	22 (95.65%)	16 (80.00%)	0.263	0.597
2	10 (14.71%)	7 (15.22%)		1 (4.35%)	4 (20.00%)		
HBsAg							
0	13 (19.12%)	13 (28.26%)	0.254	2 (8.70%)	3 (15.00%)	0.868	0.117
1	55 (80.88%)	33 (71.74%)		21 (91.30%)	17 (85.00%)		
Cirrhosis							
0	28 (41.18%)	18 (39.13%)	0.827	4 (17.39%)	6 (30.00%)	0.539	0.046*
1	40 (58.82%)	28 (60.87%)		19 (82.61%)	14 (70.00%)		
Portal hypertension							
0	29 (42.65%)	24 (52.17%)	0.317	15 (65.22%)	10 (50.00%)	0.313	0.193
1	39 (57.35%)	22 (47.83%)		8 (34.78%)	10 (50.00%)		
Tumor number							
0	38 (55.88%)	24 (52.17%)	0.697	9 (39.13%)	7 (35.00%)	0.78	0.055
1	30 (44.12%)	22 (47.83%)		14 (60.87%)	13 (65.00%)		
Tumor size							
0	33 (48.53%)	27 (58.70%)	0.286	14 (60.87%)	3 (15.00%)	0.002*	0.143
1	35 (51.47%)	19 (41.30%)		9 (39.13%)	17 (85.00%)		
BCLC							
0	12 (17.65%)	2 (4.35%)	0.156	0 (0.00%)	1 (5.00%)	0.016*	0.001*
1	25 (36.76%)	16 (34.78%)		5 (21.74%)	2 (10.00%)		
2	18 (26.47%)	16 (34.78%)		10 (43.48%)	2 (10.00%)		
3	13 (19.12%)	12 (26.09%)		8 (34.78%)	15 (75.00%)		
Tumor margin							
0	45 (66.18%)	31 (67.39%)	0.893	10 (43.48%)	10 (50.00%)	0.669	0.021*
1	23 (33.82%)	15 (32.61%)		13 (56.52%)	10 (50.00%)		
Rim enhancement							
0	28 (41.18%)	15 (32.61%)	0.354	12 (52.17%)	12 (60.00%)	0.606	0.041*
1	40 (58.82%)	31 (67.39%)		11 (47.83%)	8 (40.00%)		
Peritumoral enhancement							
0	58 (85.29%)	33 (71.74%)	0.077	19 (82.61%)	15 (75%)	0.813	0.917
1	10 (14.71%)	13 (28.26%)		4 (17.39%)	5 (25%)		

Chi-squared or Fisher’s exact tests, were used to compare the differences in categorical variables. *P<0.05 represents the statistical difference.

### The development of radiomics signature and DL signature

A total of 960 radiomics features were extracted from each phase of the MRI, and ICC was used to select 922 (96% remaining) features with high robustness (ICC>0.8). Next, mRMR and Lasso were applied to further select features, and 1, 2, 8, 5, and 1 features with rich information remained in the AP, DP, PVP, PVP_Plus3mm, and PVP_Pri3mm, respectively. Multivariate logistic regression analysis was performed using weighted summation to obtain the radiomic signature. The selected features and their relative coefficients are presented in (**Supplementary Table S2**). The distribution of radiomic signatures has shown good separability in early peritumoral recurrence. Moreover, the features of the last fully connected layer of ResNet34 were weighted to obtain a deep-learning signature (PVP_DLR). Fifteen features were selected to construct the PVP PVP-DLR model (**Supplementary Table S2**).

### Radiomics and DL signatures validation

**Figure 5:**
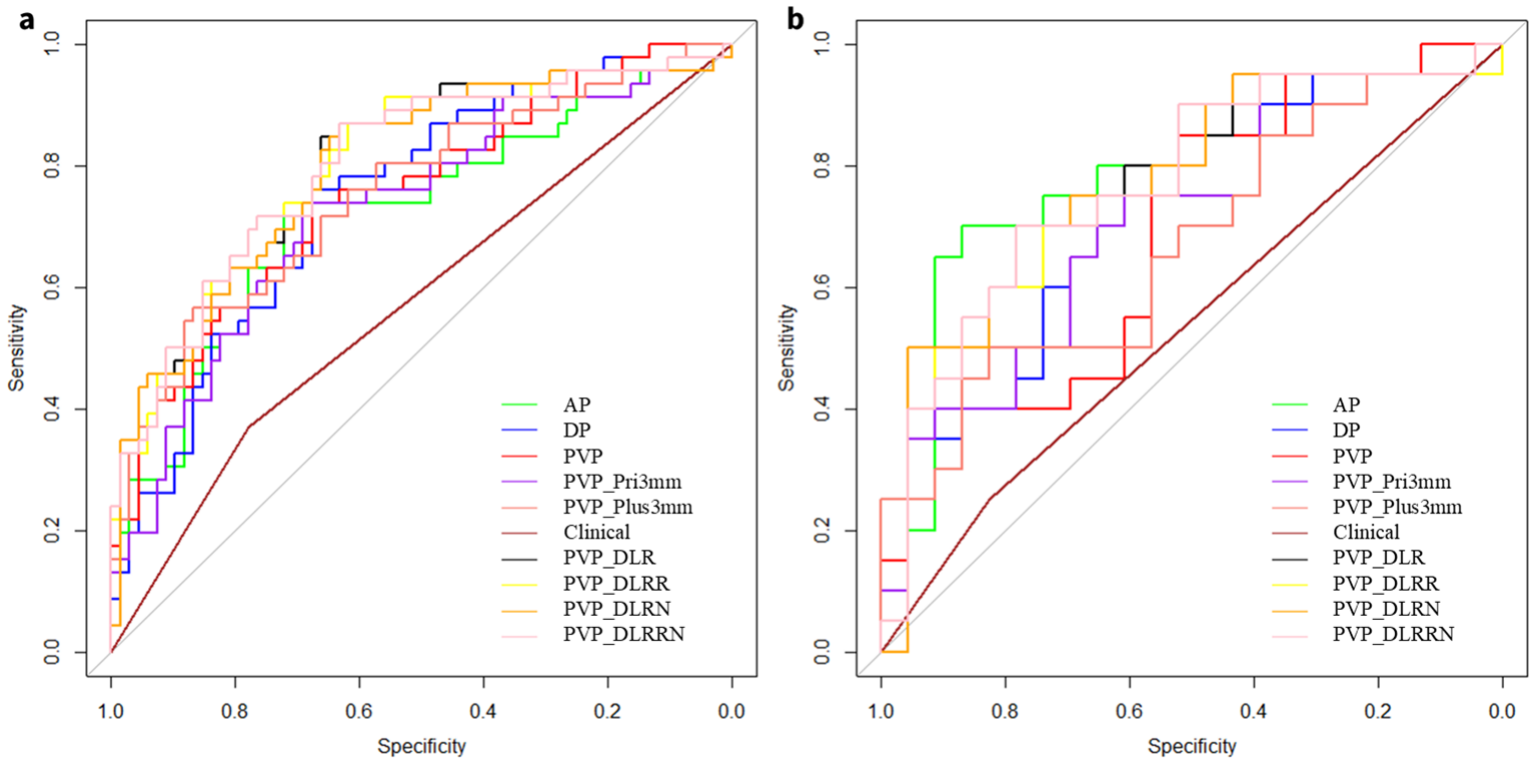
Receiver operating characteristic (ROC) curves of different models. ROC curves of AP, DP, PVP, PVP_Pri3mm, PVP_Plus3mm, clinical model, PVP_DLR, PVP_DLRR, PVP_DLRN and PVP_DLRRN model, for predicting early peritumoral recurrence (EPR) in the **(a)** Training cohort, **(b)** External validation cohort, respectively.

Five radiomics models and one deep learning radiomics model were established (**Figure 5**), and the efficacy of each model in the training and validation sets is tabulated in (**Table 2**). Among the radiomics models, the PVP model had a higher efficacy, with an AUC of 0.751 (95% CI, 0.659–0.843) in the training set and 0.691 (95%CI, 0.530–0.853) in the external validation cohort compared to the AP model, with an AUC of 0.724 (95%CI, 0.625–0.823), and the DP model with an AUC of 0.749 (95%CI, 0.659–0.839). Therefore, PVP was selected to explore intratumoral and peritumoral information, and the PVP_Pri3mm and PVP_Plus3mm models were established. The PVP_Plus3mm with an AUC of 0.754 (95%CI, 0.672–0.855) and PVP_Pri3mm model with an AUC of 0.727 (95%CI, 0.630-0.824) had comparable efficacy to the PVP model, but no significant difference (P = 0.916, P = 0.325) remained. In addition, the deep learning radiomics model based on the venous phase (PVP_DLR) had the best and most stable efficacy in the training set, with an AUC of 0.802 (0.717-0.887), and a higher efficacy in the validation set, with an AUC of 0.774 (95% CI: 0.700-0.783). The distribution of the prediction results for each model is shown in (**Supplementary Figure S2****).**

**Table 2:** Performances of the models.

	Training set (n=114)			External validation set (n=43)		
	AUC	Sensitivity	Specificity	AUC	Sensitivity	Specificity
AP	0.724 (0.625-0.823)	0.717	0.721	0.780 (0.631-0.929)	0.700	0.870
DP	0.749 (0.659-0.839)	0.761	0.676	0.722 (0.566-0.878)	0.750	0.652
PVP	0.751 (0.659-0.843)	0.739	0.676	0.691 (0.530-0.853)	0.850	0.522
PVP_Plus3mm	0.754 (0.672-0.855)	0.565	0.868	0.667 (0.501-0.834)	0.500	0.826
PVP_Pri3mm	0.727 (0.630-0.824)	0.739	0.691	0.709 (0.550-0.868)	0.750	0.609
PVP_DLR	0.802 (0.717-0.887)	0.848	0.662	0.774 (0.627-0.920)	0.700	0.783
Clinical	0.574 (0.488-0.661)	0.370	0.779	0.538 (0.413-0.664)	0.250	0.826
PVP_DLRR	0.804 (0.720-0.888)	0.870	0.618	0.767 (0.620-0.915)	0.700	0.739
PVP_DLRN	0.797 (0.711-0.882)	0.870	0.632	0.774 (0.627-0.920)	0.700	0.783
PVP_DLRRN	0.802 (0.718-0.887)	0.870	0.632	0.770 (0.623-0.916)	0.700	0.783

AP, arterial phase; DP, delay phase; PVP, portal venous phase; PVP_Plus3mm, the tumor plus peritumoral of portal venous phase; PVP_Pri3mm, the peritumoral of portal venous phase; DLR, deep learning radiomics; DLRR, deep learning radiomics and radiomics; DLRN, deep learning radiomics nomogram; DLRRN, deep learning radiomics and radiomics nomogram.

### Performance and validation of DLRRN

**Figure 6:**
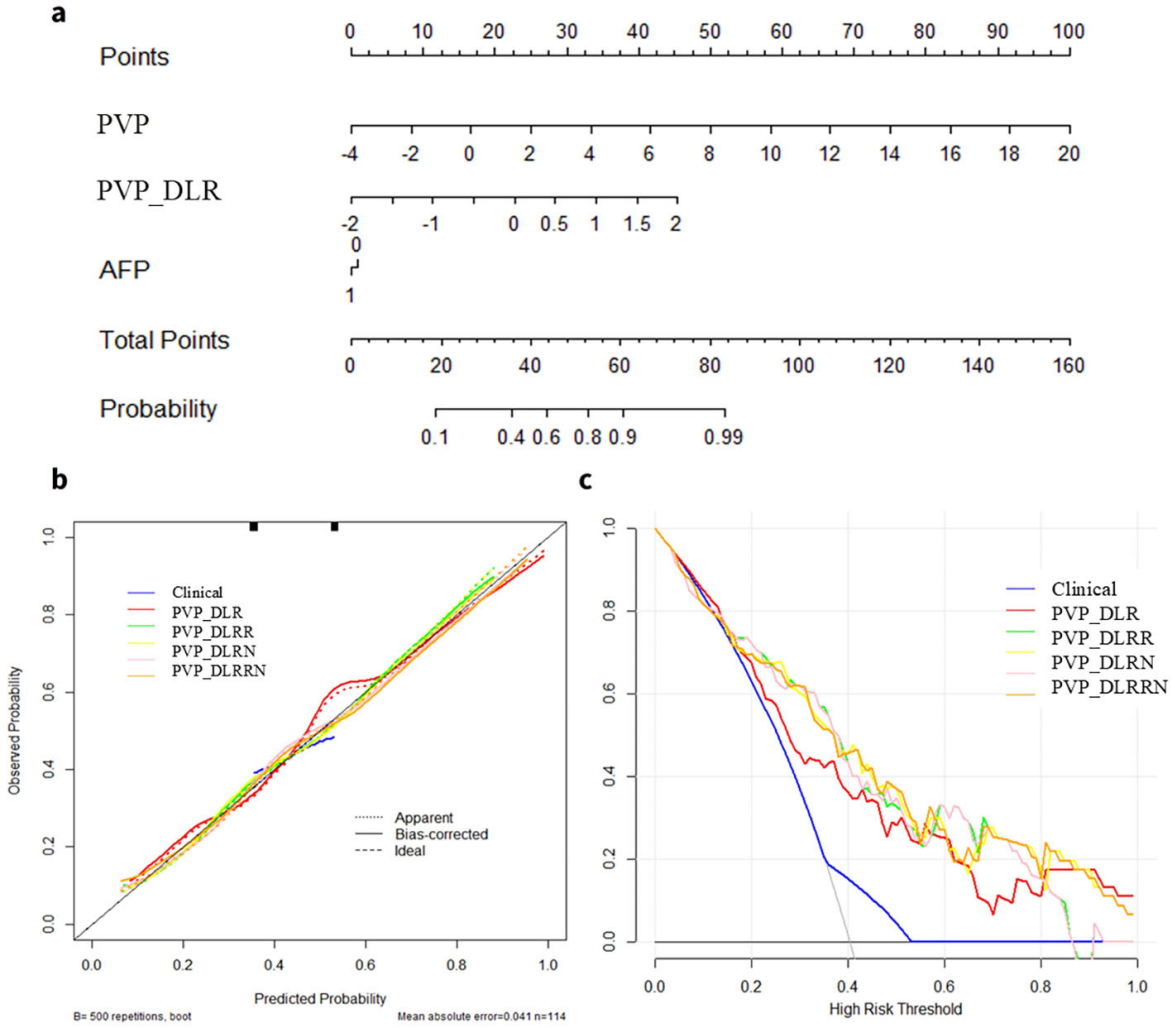
Deep learning radiomics and handcrafted nomogram (DLRRN) and their performance. **(a)** DLRRN with the handcrafted and deep learning signatures and AFP. **(b)** Calibration curves of different models with Clinical, PVP, PVP_DLR, PVP_DLRN, PVP_DLR and PVP_DLRRN in the cohorts. **(c)** Decision curve analysis for Clinical, PVP, PVP_DLR, PVP_DLRN, PVP_DLRR and PVP_DLRRN models.

In the training cohort, hand-craft based signature, DL-based signature, and AFP level were independent factors for EPR prediction using backward stepwise multivariable analysis. However, only PVP_DLR was significant, so we combined the signatures to build the PVP_DLRRN model (**Table 3**, **Figure 6a**). As shown in (**Table 2**), the performance of the combined models PVP_DLRR (AUC, 0.804), PVP_DLRN (AUC, 0.797), and PVP_DLRRN (AUC, 0.802) was not higher than that of PVP_DLR (AUC, 0.802), which was further confirmed in the external validation cohorts. There were no significant differences between the combined models (P>0.05). Furthermore, all the combined models showed significantly higher AUCs than the clinical model in the two cohorts, which also outperformed the handcrafted and DL signatures (P<0.05). NRI and IDI analyses revealed that the integration of image signatures into the DLR performed satisfactorily in the two cohorts, indicating an improved classification accuracy for the EPR prediction clinical model. The calibration curves of PVP_DLR, PVP_DLRR, PVP_DLN, and PVP_DLRRN demonstrated that the model-predicted EPR was well-calibrated with the actual observations in the cohorts(P>0.05) (**Figure 6b**). Additionally, DCA graphically indicated that the DLRRN provided a net benefit over other models over the relevant threshold range in the entire cohort (**Figure 6c**). (**Figures 5**, **6**) shows correctly classified examples from the EPR and non-EPR, respectively.

**Table 3:** Related factors for EPR prediction in HCC.

Intercept and variable	β	OR (95%CI)	P
Intercept	0.08493	–	0.7811
AFP	-0.00971	0.907 (0.326-2.530)	0.853
PVP	0.43748	1.549 (0.831-2.888)	0.168
PVP_DLR	1.19241	3.295 (1.377-7.886)	0.007*

β is the regression coefficient. *p<0.05.

## Discussion

In this study, we constructed various radiomics models of intratumoral, peritumoral, and intratumoral combined peritumoral derived from CE-MR images and deep learning radiomics models derived from the venous phase to predict the early peritumoral recurrence of DEB-TACE in patients. We confirmed that the performance of PVP was comparable to that of PVP_Pri3mm and PVP_Plus3mm. Furthermore, a combined nomogram incorporating the clinical factors AFP, PVP rad-score, and DLR rad-score exhibited excellent and stable performance in recurrence prediction compared to the clinical model.

Few studies have predicted the response to DEB-TACE in patients with HCC. Some textural features, such as entropy and skewness, were found to be able to identify responders (22). In terms of radiomics, a limited number of previous studies have focused on the application of CT-based features prior to DEB-TACE overall survival, displaying moderate performance with AUCs of 0.70-0.76 (23, 24). Nevertheless, the current two radiomics studies both focused on CT images and survival outcomes, which could not achieve an earlier prediction to guide DEB-TACE. However, the findings were of limited clinical relevance because of the relatively small sample size and lack of validation in multicenter cohorts. Intriguingly, most of the features selected in the radiomics signature were transformation factors in the current study, especially the Laplacian of Gaussian (LoG) and wavelet-based features, providing more detailed information about tumor heterogeneity.

The PVP model with an AUC of 0.751 (95%CI, 0.659-0.839) showed better efficacy than the AP and DP models with AUC of 0.724 (95%CI, 0.625-0.823), 0.749 (95%CI,0.659-0.839). Several recent studies have investigated the efficacy of CT radiomic models for early and late recurrence after hepatocellular carcinoma resection, with moderate to good results and AUCs of 0.749-0.870 (25), respectively. However, 3D-ROI segmentation and independent external validation may result in a statistical danger. In addition, the AP performed better in the external validation set (AUC = 0.780), which is consistent with the findings of Li et al (26). Normal liver parenchyma derives its main blood supply from the portal vein, whereas typical HCC is mainly supplied by the hepatic artery, and this difference in blood supply contributes to the imaging characteristics of HCC on enhanced MRI.

Furthermore, to capture relevant features of the microenvironment surrounding the tumor and explore potential links between this and tumor biological behavior, the PVP_Plus3mm and PVP_Pri3mm models were established, and their performance was comparable to that of the PVP model (P>0.05), consistent with the results of Song and Kim et al (27, 28). Microvascular invasion (MVI) is a histopathological diagnosis used to characterize cancerous thrombus formation within tiny blood vessels surrounding a tumor. MVI in HCC is mostly found in the tiny branches of the portal vein in the tissues surrounding the tumor, which is one of the important manifestations of tumor microinvasion and micrometastasis, and is closely related to early recurrence after HCC treatment. Zhang et al. found that a radiomics model based on preoperative 5 mm T1WI-MR images of the surrounding tumor performed poorly in predicting HCC recurrence after radiofrequency ablation (29). In contrast, our study showed that the efficacy of PVP_Pri3mm is comparable to that of PVP and PVP_Plus3mm, suggesting that the 3 mm peritumor radiomic profile may include abundant information related to the microenvironment surrounding the tumor. A possible reason for this may be that 60.47% of the tumors had a diameter greater than 50 mm.

In this study, a DL method based on the ResNet-34 architecture was applied for DL feature extraction. Notably, unlike handcrafted features, the DL method does not require slice-by-slice segmentation, which not only reduces the contour variability of manual segmentations but also enhances efficiency. Moreover, DL provides in-depth information, including specific tasks in the neural network hidden layers without predefined features. The features captured by the DL algorithm can predict lymph node metastasis (30, 31), neoadjuvant chemotherapy response in gastric cancer (16). The DL signature in our study presented a promising performance in EPR prediction with AUCs of 0.802 and 0.774, higher than that in the previous study predicting early recurrence after HCC surgery based on preoperative CT images using DL features with an AUC of 0.723 (32). Moreover, the DL prediction model outperformed the handcrafted signature and clinical models in terms of discrimination ability in both training and validation cohorts. These results indicate that DL offers a wealth of information that reflects the spatial heterogeneity of tumors.

Furthermore, the combined PVP_DLRR, PVP_DLRN, and PVP_DLRRN models were established in this study, and the prediction ability of the models was far better than that of the clinical model in the cohorts (P<0.05). Previous studies have indicated that various clinical or molecular risk factors are associated with TACE response. However, these metrics were inconsistent across all studies. The BCLC and tumor size were significant in the validation cohort, but no significance was found in the training cohort. Considering that the small sample size may have resulted in statistical bias, the AFP, which is significantly different in the training cohort, was incorporated into our clinical model. Specifically, the AUC of the clinical model was only 0.538 in the external cohort, which was significantly lower than those of the other models. Additionally, clinical factors are specific aspects of tumors. The patients with similar features exhibited different responses. This may explain the poor performance of the clinical model in different patient distributions. DLRN mines high-dimensional imaging features, followed by the comprehensive quantification of intratumor heterogeneity, thereby improving performance.

This study has several limitations. First, due to its retrospective design, the sample size was limited, and pathological results were unavailable, potentially introducing selection bias and uneven distribution of patients’ clinical data. However, we mitigated this by incorporating a multicenter cohort and applying strict inclusion criteria. Future work should involve well-designed prospective studies, larger datasets, and robust regularization methods to validate the model’s generalizability and clinical utility. Second, because deep learning (DL) features are abstract “black-box” features, our interpretability analysis remains insufficient. In follow-up studies, we plan to employ visualization tools (e.g., Grad-CAM and LIME) to identify tumor regions of model focus and correlate DL features with pathological mechanisms, thereby enhancing the model’s clinical trustworthiness and applicability. Additionally, although we evaluated intraclass correlation coefficients (ICCs), discrepancies persist due to the time-intensive process and inherent inter-observer variability in manual, layer-by-layer tumor delineation. Future clinical applications will require automated and reliable segmentation methods, such as those described in the literature (33, 34). In subsequent studies, we intend to integrate these automated techniques to boost efficiency, reproducibility, and minimize biases. Nevertheless, our research pioneered a deep learning radiomics model for predicting early peritumoral recurrence after DEB-TACE, demonstrating superior efficacy.

## Conclusion

In conclusion, the DLR based on preoperative MRI could be a new prognostic hallmark of HCC in patients undergoing DEB-TACE. The prognostic model DLRRN based on DLR-score and handcraft-score nomogram may accurately predict EPR, which may improve the assessment of preoperative treatment selection and postoperative prognosis of HCC patients.

## Data Availability

The datasets presented in this study can be found in online repositories. The names of the repository/repositories and accession number(s) can be found in the article/**Supplementary Material**.
