# Supplementary material for: Deep-learning radiomics and hand-crafted radiomics utilizing contrast-enhanced MRI to predict early peritumoral recurrence after DEB-TACE with hepatocellular carcinoma: a two-center study
Source: Front Oncol. 2025 Nov 18;15:1642828. doi: 10.3389/fonc.2025.1642828 (PMC12669013; doi:10.3389/fonc.2025.1642828)
Supplement: Supplementary file 1 [file DataSheet1.docx]

**Clinical Characteristics**

Routine preoperative clinical data and laboratory examinations were collected through the electronic medical record system (EMRS) of the Second Affiliated Hospital of Chongqing Medical University and the Southwest Hospital of the Army Military Medical University. The clinical characteristics of the patients including: (1) General data: gender, age, History of hepatitis B and cirrhosis; (2) laboratory tests within one week before DEB-TACE: alpha-fetoprotein (AFP), alanine aminotransferase (ALT), aspartate aminotransferase (AST), albumin (ALB), prothrombin time (PT), Child-Pugh class, and BCLC B subclassification (B1–B4); (3) tumor characteristics, such as number, size, margin, rim enhancement and peritumoral enhancement. Continuous variables were categorized into categorical variables according to the thresholds.

**DEB-TACE procedure**

DEB-TACE procedure was performed on all patients by three interventional radiologists (16 years, 10 years and 20 years of interventional experience) in four digital subtraction angiography (DSA) rooms. Before the operation, The CalliSpheres® Beads (Jiangsu Hengrui Medicine Co. Ltd., China) with diameters of 100–300 µm or 300–500 µm loaded with 30–60 mg pirarubicin. First, under local anesthesia, the right femoral artery was incubated by the modified Seldinger technique, a 5F RH catheter (Terumo Corporation, Tokyo, Japan) was catheterized into the celiac trunk, superior mesenteric artery or common hepatic arteriography to detect the tumor-supplying vessels. The tumor-feeding vessels were super selectively cannulated with a 2.4F microcatheter (Merit Maestro, Merit Medical System, Inc., Utah, USA) after confirmation, and when the microcatheter tip was in the proper position, the DEBs were injected slowly through the microcatheter. The embolization was stopped when the tumor staining completely obstructed. Finally, angiography was performed to confirm whether the tumor had a residual blood supply.

Table S1. The MR protocols of the two centers

| Center | Sequence | Strength | TR(ms) | TE(ms) | FOV(mm²) | matrix | slice thickness(mm) | slice spacing(mm) |
| --- | --- | --- | --- | --- | --- | --- | --- | --- |
| Center 1  (The Second Affiliated Hospital of Chongqing Medical University) | T1WI | 1.5T Avanto | 120 | 4.76 | 345x379 | 288x  262 | 7 | 9.1 |
|  |  | 1.5T HDXT2012 | 220 | 4.7 | 320x320 | 512x512 | 8 | 9 |
|  | T2WI | 1.5T Avanto | 2500 | 108 | 288x262 | 192x192 | 7 | 8.4 |
|  |  | 1.5T HDXT2012 | 2820 | 92.768 | 320x320 | 512x512 | 7.5 | 8.5 |
|  | DWI | 1.5T Avanto | 4300 | 72 | 308x379 | 384x312 | 7.5 | 9.75 |
|  |  | 1.5T HDXT2012 | 4495 | 72.5 | 400x400 | 256x256 | 8 | 9 |
|  | T1WI-CE | 1.5T Avanto | 3.91 | 1.44 | 284x379 | 288x216 | 3 | 0 |
|  |  | 1.5T HDXT2012 | 3.524 | 1.688 | 320x320 | 512x512 | 5 | 2.5 |
| Center 2  (The First Affiliated Hospital of Army Military Medical University) | T1WI | 3.0T uMR 770 | 153.6 | 2.48 | 336x319 | 384x307 | 5 | 6.5 |
|  |  | 3.0T TrioTim | 206 | 3.83 | 320x154 | 256x168 | 6 | 7.8 |
|  | T2WI | 3.0T uMR 770 | 900 | 94.24 | 384x364 | 640x506 | 5 | 6.25 |
|  |  | 3.0T TrioTim | 700 | 88 | 320x195 | 320x244 | 6 | 7.8 |
|  | DWI | 3.0T uMR 770 | 3000 | 66 | 144x105 | 288x242 | 5 | 6.5 |
|  |  | 3.0T TrioTim | 5500 | 81 | 180x109 | 180x136 | 5 | 6.5 |
|  | T1WI- CE | 3.0T uMR 770 | 3.36 | 1.51 | 320x240 | 384x268 | 2.5 | 2.5 |
|  |  | 3.0T TrioTim | 3.42 | 1.25 | 320x154 | 320x220 | 2.5 | 0 |

Table S2. Results of radiomics feature selection and signature building

| **Rad-score** | **Features** | **Coefficient** |
| --- | --- | --- |
| AP | intercept | -1.46998 |
|  | wavelet.LHH_gldm_LargeDependenceHighGrayLevelEmphasis | 0.000208 |
| DP | intercept | -0.74386 |
|  | original_shape_MajorAxisLength | 0.020112 |
|  | wavelet.HHH_gldm_LowGrayLevelEmphasis | -2.3893 |
| PVP | intercept | -1.0147 |
|  | wavelet.HLH_glszm_GrayLevelNonUniformity | -0.00071 |
|  | original_glszm_SmallAreaLowGrayLevelEmphasis | -105.222 |
|  | wavelet.HHH_glszm_ZoneVariance | 2.58E-10 |
|  | log.sigma.5.0.mm.3D_gldm_DependenceVariance | 0.035365 |
|  | log.sigma.5.0.mm.3D_glszm_LargeAreaHighGrayLevelEmphasis | 1.68E-08 |
|  | log.sigma.5.0.mm.3D_glszm_ZoneVariance | -1.41E-06 |
|  | original_gldm_LargeDependenceHighGrayLevelEmphasis | 4.00E-06 |
|  | wavelet.HHL_glszm_LargeAreaHighGrayLevelEmphasis | 4.53E-10 |
| PVP_Plus3mm | intercept | -0.06127 |
|  | original_shape_Sphericity | -2.90128 |
|  | wavelet.HLL_firstorder_Maximum | 0.006833 |
|  | log.sigma.3.0.mm.3D_firstorder_Maximum | 0.003935 |
|  | log.sigma.5.0.mm.3D_glszm_LargeAreaHighGrayLevelEmphasis | 4.29E-09 |
|  | wavelet.HHL_glszm_LargeAreaLowGrayLevelEmphasis | 7.09E-06 |
| PVP_Pri3mm | intercept | -1.14848 |
|  | wavelet.LHL_gldm_DependenceNonUniformity | 0.000378 |
| PVP_DLR | intercept | -0.21453 |
|  | X_206 | 0.02791 |
|  | X_340 | 1.96161 |
|  | X_221 | -0.22502 |
|  | X_160 | 0.16504 |
|  | X_228 | 0.37057 |
|  | X_203 | 0.18441 |
|  | X_381 | 0.43620 |
|  | X_456 | -0.39865 |
|  | X_370 | 0.39603 |
|  | X_319 | 0.77904 |
|  | X_85 | -0.28357 |
|  | X_358 | 0.20885 |
|  | X_21 | -0.27605 |
|  | X_211 | -0.40659 |
|  | X_101 | -0.17989 |


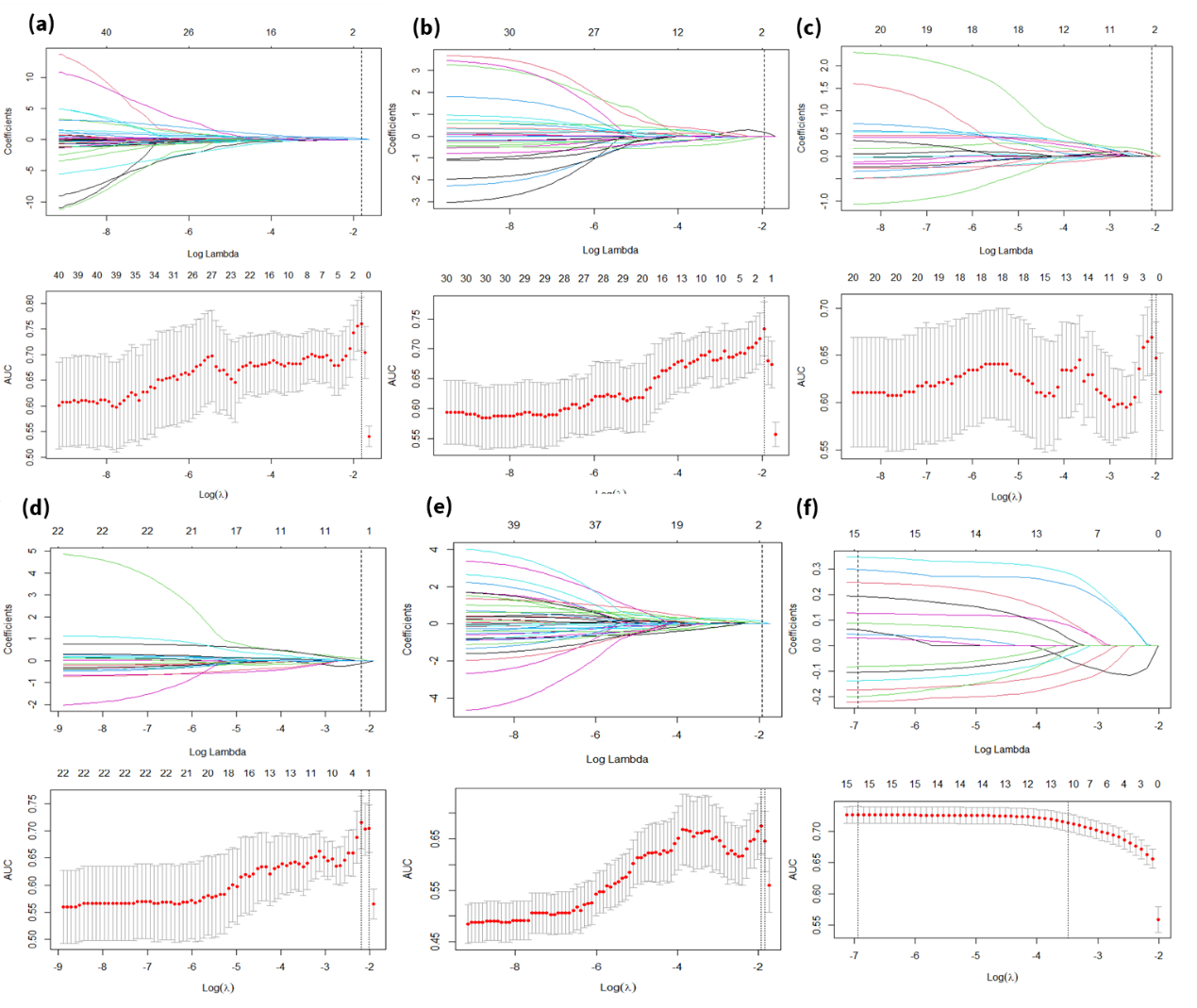


Fig. S1 Lasso algorithms. (a) AP; (b) DP; (c) PVP; (d) PVP_Plus3mm; (e) PVP_Pri3mm; (f）PVP_DLR


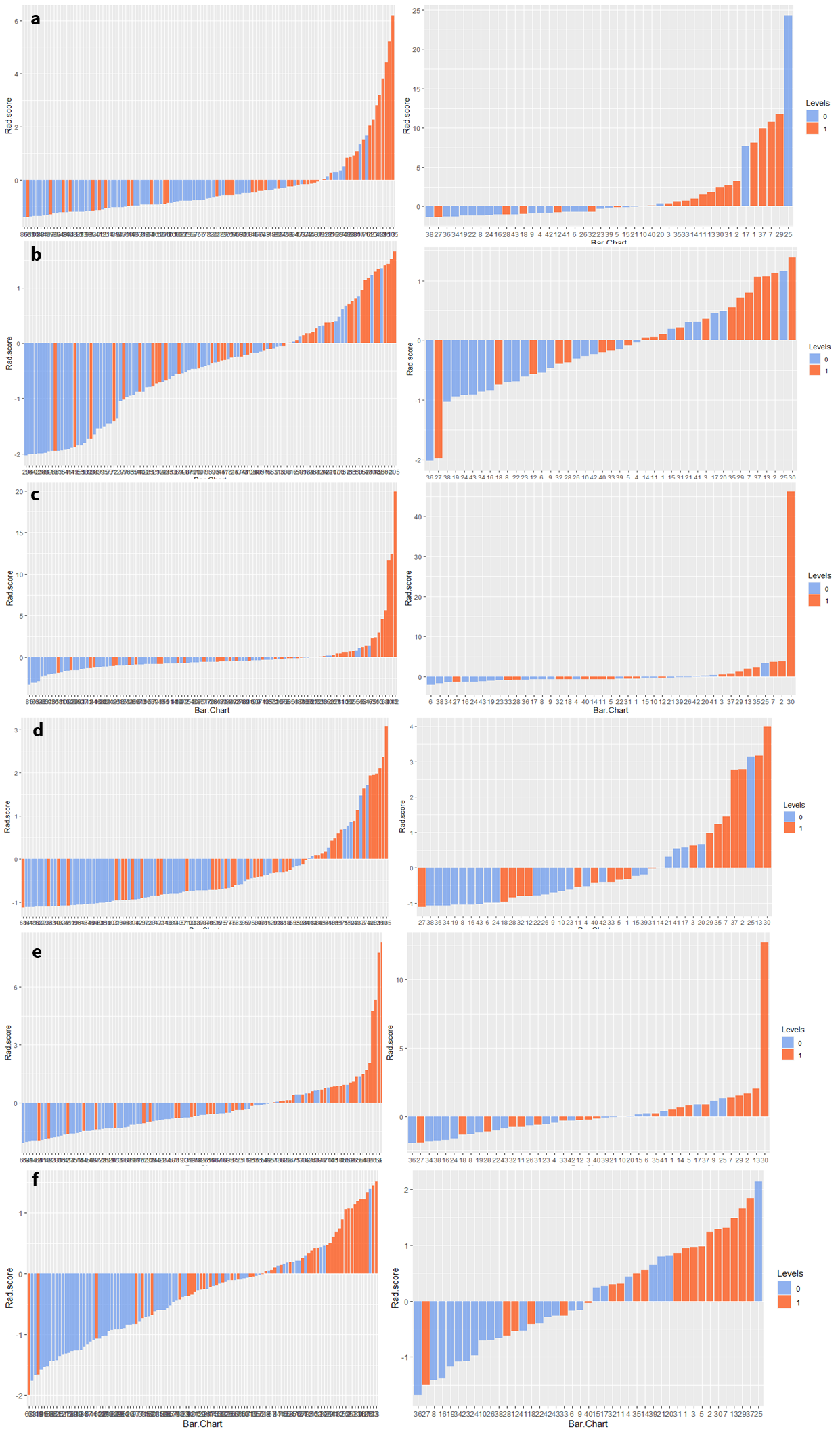


Fig. S2 Bar chart of different models in the training and validation cohort. (a) AP; (b) DP; (c) PVP; (d) PVP_Pri3mm; (e) PVP_Plus3mm; (f）PVP_DLR.
